# Clinical Significance of the Interaction between Human Papillomavirus (HPV) Type 16 and Other High-Risk Human Papillomaviruses in Women with Cervical Intraepithelial Neoplasia (CIN) and Invasive Cervical Cancer

**DOI:** 10.1155/2020/6508180

**Published:** 2020-10-26

**Authors:** Arsenio Spinillo, Mattia Dominoni, Anna C. Boschi, Cecilia Sosso, Giacomo Fiandrino, Stefania Cesari, Barbara Gardella

**Affiliations:** ^1^Department of Obstetrics and Gynecology, Fondazione IRCCS Policlinico San Matteo, University of Pavia, Pavia 27100, Italy; ^2^Department of Pathology, Fondazione IRCCS Policlinico San Matteo, Università of Pavia, Pavia 27100, Italy

## Abstract

The aim is to evaluate the clinical consequences of coinfection between HPV 16 and other high-risk HPVs among women with a histological diagnosis of CIN or invasive cervical cancer. A total of 2985 women, with a diagnosis of either CIN or cancer (<IB) on cervical or cone biopsy, were included. HPV genotypes were identified using the INNO-LiPA HPV genotyping assay, version EXTRA, on cervical scraping, before the colposcopic evaluation and the colposcopic biopsies or conization. In the overall population, HPV16 interacted positively with HPV18 (RR = 2, 95% CI 1.5–2.6) and negatively with HPV33, 51, 52, and 66, in log-linear analysis. There was an excess of CIN3 diagnoses among subjects coinfected with HPV16 and HPV18 or HPV52, although the absolute number of cases was relatively small. In a logistic model, the odds ratio of CIN3+ associated with coinfection of HPV16 and HPV18 (OR = 3.8, 95% CI 2.5–5.7, *p*=0.004 compared to single HPV16) or HPV52 (OR = 3.6, 95% CI 2.6–5.1, *p*=0.009 compared to single HPV) was higher than that associated with single HPV 16 infections. Finally, multiple infections had no effect on residual disease and did not influence the recurrence of high-grade CIN during a median follow-up of 25 months (IR 17–41). HPV16 interacted positively with HPV18 and negatively with HPV33, 51, 52, and 66 supporting the notion that HPV16 interacts mostly negatively with other HR-HPVs in CIN lesions. Among specimens coinfected with HPV16 and 18 or 52, there was an excess of CIN3+ although the impact on the prevalence of severe cervical lesions was limited.

## 1. Introduction

Multiple simultaneous high-risk (HR) human papillomavirus (HPV) infections among women with abnormal cervical cytology or histology is common ranging from 20 to more than 50% of cases [1, 2]. Several factors influence coinfection including the type of population studied (Pimenoff et al. demonstrated that the differential geographic clustering patterns derived from the ancient migration from Africa to Eurasian, during the human evolution, [3]), younger age, type of specimen (cytology vs histology), recent sexual history, human immunodeficiency virus (HIV) infection, or sensitivity of the genotyping system used [4, 5]. Several cross-sectional studies suggest that multiple HR-HPV infections are associated with an increased risk of severe CIN [6, 7] although others failed to confirm these findings [8]. Longitudinal data based both on cytologic and histological findings also suggest that multiple HPV infections are associated with worse colposcopic pictures and increased rates of progression or recurrence after treatment [5, 9–11]. Biologically, it has been demonstrated that simultaneous infections of a single cell by multiple HPV types is possible and that mechanisms of interference on replication or superinfection exclusion could regulate subsequent detection of viruses and their oncogenic activity [12, 13].

Most of the published population data on the role of coinfection relating to the severity of CIN and to the recurrence after treatment are based on multiple infections as a whole rather than on the interaction of single HPV types. The purpose of the present study was to evaluate the rates and the prognostic significance of coinfection between HPV16 and other HR-HPV types in a population of women with biopsy-proven cervical intraepithelial neoplasia or invasive cervical cancer.

## 2. Patients and Methods

Data for this retrospective cohort study were extracted from a database containing prospectively collected clinical, colposcopic, and virological details from subjects attending the colposcopic unit of our department because of an abnormal pap smear in the period 2010–2018. The database was composed by a series of anamnestic items gathered after a structured interview at entry and by clinical, colposcopic, and virological features compiled at entry and during the follow-up. Subjects (from 21 to 65 years of age) were referred to the cytologic screening unit of our department, from private practice and from screening services of external institutions. Exclusion criteria included pregnancy, HPV test or treatment for CIN in the year before enrollment, total hysterectomy, and the use of vaginal medication in the previous 3 days before screening. In the study, only subjects with a histological diagnosis of CIN or invasive cervical cancer (<stage IB) based on colposcopic biopsies or histological examination of specimens from loop electrosurgical procedures (LEEP) or cold-knife conizations were included. The study was approved by the local Ethical Committee of our hospital, and patients gave their informed written consent (RCXXX). All patients were treated according to an established protocol including HPV DNA detection and typing and colposcopy with targeted biopsies. Cervical samples for HPV typing were obtained immediately before colposcopy. After speculum examination, scrapes were taken with a cervical brush, suspended in Thin Prep-PreservCyt Solution (Cytic Corporation, Marlborough, MA, USA), and stored at 4°C. DNA extraction was performed by lysis and digestion with proteinase K. HPV sequences from the L1 region were amplified by polymerase chain reaction (PCR) using SPF10 primers in a 50 *μ*l final reaction volume for 40 cycles. Appropriate positive and negative controls were introduced for each set of reactions. Concurrent amplification of beta globin sequences was used as a control for DNA adequacy. HPV type-specific sequences were detected by the line probe assay INNO-LiPA HPV genotyping assay, version EXTRA (Fujirebio Europe. Gent, Belgium), according to the manufacturer's instructions. The EXTRA version of the assay allows the simultaneous and separate detection of 18 high-risk (HR) HPV types (HPV16, 18, 31, 33, 35, 39, 45, 51, 52, 56, 58, and 59) with proven carcinogenicity and Group I of the classification of International Agency for Research on Cancer (IARC), HPV26, 53, 66, 68, 73, and 82 with probable carcinogenicity and Group II IARC classification, seven low-risk HPV types (6, 11, 40, 43, 44, 54, and 70), and two unclassified-risk HPV types (69/71 and 74) [14]. Hybridization patterns were automatically analyzed by the LiRAS system and checked by two independent readers.

A standardized colposcopic examination was performed immediately after cervical brushing for HPV typing by two gynecology residents certified by the Italian Society of Colposcopy. Multiple-targeted cervical biopsies were obtained in all cases where CIN was suspected on colposcopy and in all cases of high-grade squamous cervical lesions (HSIL) irrespective of colposcopic impression. Endocervical curettage was performed, according to the judgment of the clinician, when the extent of the lesion or the cervical squamocolumnar junction was not entirely visible or in the case of the presence of atypical glandular cells (AGC) on Pap smear. Histological diagnoses were based on consensus decision of two expert gynecological pathologists. In order to analyze the data, we either used the histological diagnosis obtained from punch biopsy or, in more severe cases, the diagnosis was made after cone biopsy obtained from LEEP (loop electroexcision procedure) or cold-knife excision. After diagnostic workup and treatment, patients referred to the cytologic screening unit of our department were enrolled in a follow-up program including the following:

- Observation, colposcopy, and/or cytology every 6–12 months for subjects with negative colposcopic impression and/or negative histological findings after an abnormal Pap smear
- Observation, colposcopy, and cytology every 6 months for CIN1 lesions
- HPV testing coupled with colposcopy and cytology every 6 months for treated CIN2–3 or persistent CIN 1 lesions.

Univariate statistical analysis was carried out with the Kruskal–Wallis analysis of variance and chi-squared test to compare categorical variables, respectively. The Bonferroni method for multiple comparisons was used to evaluate partitioned chi-squared tests in multiway contingency tables. Spearman rank correlation coefficient was used to test for linear trend. To evaluate the likelihood of coinfection between HPV16 and other high-risk HPV types, we used log-linear analysis. An automated hierarchical stepwise method was used to select the model containing the least number of significant interactions necessary to fit the observed tables of associations between HPV16 and other HR-HPVs. In this model, nonsignificant interactions between variables are progressively eliminated (backward elimination) from the saturated model based on the likelihood ratio of chi-square. An initial model contained 18 HR-HPV types (HPV16, 18, 31, 33, 35, 39, 45, 51, 52, 56, 58, and 59 and three with probable carcinogenicity (HPV26, 53, and 66). The model fit was assessed by deviance modification. We used both ordered and multinomial/binomial logistic regression analysis with the aim of testing the association of HPV coinfection on the incidence and severity of cervical intraepithelial neoplasia. In ordered logistic regression equations, the outcome was modeled as a 4-level categorical variable (CIN1, CIN2, CIN3, and invasive cancer), whereas exposure variables included age (continuous), HIV status (yes, no), parity (yes, no), cigarette smoking at entry (no, <10 cig/day, ≥10 cig/day), contraceptive use (no, hormonal, barrier, IUD), and HPV status in 6 categories (baseline HPV16 and HPV interacting negative; HPV16 and HPV interacting positive; single HPV 16 infection; single HPV interacting infection; multiple infections associated with HPV16 (interacting HPV negative); multiple infections associated with HPV interacting (HPV 16 negative)) We fitted the model estimating partial proportional odds using the gologit2 algorithm for the equations with variables violating the parallel regression assumption of ordered data [15]. Multinomial/binary logistic regression models contained the same explanatory variables of ordered regression. Regression coefficients obtained from multinomial/binomial logistic equations were compared using the Wald test. All analyses were carried out using STATA 13.0 [16].

## 3. Results

During the period of the study, a total of 3601 subjects between 21 and 65 years of age, with an abnormal pap smear who attended our department for colposcopic evaluation, were initially included in the study. Targeted biopsies were not taken in 373 (10.4%) women (325 subjects with Atypical Squamous Cell of Undetermined Significance (ASCUS) and 48 with low grade SIL) with entirely negative colposcopic examination; these women were excluded from the analysis. In addition, from our study, we excluded 231 (6.4%) subjects with negative results on biopsies (104 with ASCUS, 82 with LSIL, 8 with AGUS, and 37 with HSIL/ASCH) and 12 (0.3%) subjects with macroscopic invasive cervical cancer (>Stage IB) who did not necessitate conization for evaluation of invasion which left us with 2985 subjects for our final analysis (Figure Supplementary materials 1).

The sociodemographic, cytologic, and virological data of the population under investigation is reported in Table 1. Overall, the rates of single HR, multiple HR, and low-risk HPV infections were 48.2% (1440/2985), 30.7% (915/2985), and 7.5% (225/2985), respectively. The HPV test was negative in 391 (13.1%) and undetermined in 14 (0.5%) additional subjects. Multiple HR infections included 2 and 3 or more viruses in 780 (26.1%) and 135 (4.5%) subjects, respectively. There was a significant and direct linear trend relating age and HPV16, HPV18, HPV31, HPV33, HPV45, and HPV52 to an increase in the severity of cervical disease (*p* < 0.05 for all the comparisons). Overall, after adjustment for age, parity, HIV infection, and type of contraceptive used, the ORs of CIN2, CIN3, and invasive cancer were 2.1 (95% CI = 1.5–2.8), 3.4 (2.51–4.7), and 4.8 (21.7–10.8) for single and 2.3 (95% CI = 1.6–3.2), 5.4 (3.9–7.4, *p* < 0.001 compared to single infection), and 5.7 (2.5–13.2) for multiple infections, respectively.

To test the significance of simultaneous associations of HPV16 with other HR-HPVs, we used hierarchical log-linear models. HR-HPVs were inserted in the model and backtested for simultaneous associations. The K-way and higher order effects test suggested that two-way interactions fitted the model adequately (*p* < 0.001 for two-way interactions, *p*=0.4 for three-way interactions). The results of modeling are reported in Table 2. In low-grade lesions, only HPV52 interacted negatively with HPV16. In the overall population, HPV16 interacted positively with HPV 18 (observed vs expected rate of coinfection = 99/2985 as compared to 67/2985). On the other hand, HPV16 interacted negatively with HPV51 (observed vs expected rate of coinfection = 85/2985 as compared to 111/2985), HPV52 (observed vs expected rate of coinfection = 157/2985 as compared to 195/2985), and HPV66 (observed vs expected rate of coinfection = 33/2985 as compared to 58/2985).

In stratified analysis, single HPV16 infections were associated with a higher than expected rate of CIN3 and invasive cancer. Among subjects coinfected with HPV16 and HPV18 (observed vs expected rate of CIN3 = 45/678 as compared to 22/678, 3.4% of CIN3 cases) or HPV16 and HPV52 (observed vs expected rate of CIN3 = 65/678 as compared to 37/678, 4.1% of CIN3 cases), there was a statistically significant excess of CIN3 diagnoses, whereas the rates of coinfection with HPV16 and HPV51 or HPV66 were similar in the four groups of severity of cervical disease (Table 3).

To evaluate the effect of coinfection of HPV16 with HPV18 and HPV52 on the prevalence of CIN correcting for potential confounders, we used both ordinal and multinomial logistic analyses. In ordinal equations, outcome was inserted as a 4-level (CIN1, CIN2, CIN3, and cancer) ordinal variable (Table 4). Overall, in ordinal models, multiple HPV 16 infection was associated with an increased severity of cervical disease (OR: 5, 95% CI = 4.6–5.4) compared to single HPV 16 infections (OR = 2.5, 95% CI = 2.2–2.9, *p* < 0.001 compared to multiple infection). Comparing odds ratios (Table 4), coinfection of HPV16 and HPV18 or HPV52 was associated with an increased severity of cervical disease in contrast to single HPV16 infections (*p*=0.011 and *p*=0.04) or multiple HPV16 infections without HPV 18 (*p*=0.003) or HPV 52 (*p*=0.001).

In multinomial logistic equations, the outcome was modeled as a 3-level nominal variable (low-grade CIN, high-grade CIN, and cancer). The likelihood of high-grade CIN associated with HPV16/18 and HPV16/52 coinfection was higher than those associated with single HPV16 (*p*=0.008 and *p*=0.03, respectively) or multiple HPV16 without HPV18 (*p*=0.01) or HPV52 (*p*=0.028) (Table 4). The overall risks of high-grade CIN and invasive cancer were 2.7 (95% CI = 2.1–3.4) and 3.8 (95% CI = 3–4.8) among single infections and 4.9 (95% CI = 2.17–10.9, *p*=0.01 compared to single HPV16 infections) and 5.73 (2.5–13.2, *p*=0.46 compared to single HPV16 infections) among multiple HPV16 infections. When the outcome was modeled as CIN3+ subjects vs others, the odds ratio of CIN3+ was higher among women coinfected with HPV16 and HPV18 *p*=0.004 compared to single HPV16 infection) or coinfected with HPV16 and HPV52 (*p*=0.009) than women infected only with HPV16 (Table 4).

Of the 2985 enrolled subjects, a conization procedure (1259 LEEP and 42 cold-knife) was performed in 1301 (43.6%) of the cases (Figure S1). Indications for conization included 534 (41%) subjects with persistent CIN1, 748 (57.5%) with CIN2+ and 19 (1.5%) with microscopic cervical cancer on cervical biopsies. Sixty-four (4.9%) subjects with CIN1 (50 cases) or CIN2 (14 cases) on punch biopsy had negative histology on cone but were included in the analysis with their original histological diagnoses.

The rates of low- and high-grade residual disease after conization on either ectocervical and endocervical margins are reported in Table S5). The rates of high-grade (CIN2+) residual disease on cone were 154 (11.8%) on ectocervical, 7 (0.5%) on endocervix, and 91 (7%) in both areas. Overall, the rates of high-grade residual diseases were 13.5% (24/178) in low-risk/negative HPV, 18.3% (121/661) in single HR, and 23.2% in multiple HR-HPV infection (107/462*p*=0.015 compared to low-risk negative HPV). In stratified analysis, the coinfection between HPV16 and either HPV18, 33, 51, 52, or 66 did not influence the rates of residual disease on endocervical and ectocervical margins.

Out of the 1301 women who received conization, follow-up data (follow-up of at least 6 months) were available in 1090 subjects (83.8%) mainly enrolled in the cytologic screening unit of our department. Subjects with invasive cervical cancer were excluded from the follow-up analysis. The median follow-up time for the entire cohort was 25 months (IQR 17–41 months) with a median of 3 colposcopies (IQR 1–5). A biopsy-confirmed low-grade and high-grade CIN after the first 6 months of follow-up was diagnosed in 94 (8.6%) and 46 (4.2%) of the subjects, respectively. The persistence/recurrence rate of high-grade CIN (CIN2+) was uninfluenced by multiple HR-HPVs upon enrollment. In fact, the recurrence rate was 0.1 cases per woman/year (4 cases out of 151) among subjects with low-risk/negative HPV, 1.7 cases per woman/year (25 cases out of 580), and 1.8 cases per woman/year (17 cases out of 355) among HPV16 single and multiple infections (*p*=0.08), respectively. Persistence/recurrence rate was also uninfluenced by coinfection between HPV16 and HPV18. The recurrence rate was 1.3 cases per woman/year in subjects with single HPV16 infection (7/197) and 2 cases (2 cases out of 41) per woman/year among subjects coinfected by HPV16 and HPV18. Finally HPV 16 persistence (HPV 16 positivity at any follow-up visit among subjects already positive at entry) was 52.3% (103/197) among single and 44.2% (46/104) among multiple HR-HPV16 infection at entry (*p*=0.22).

## 4. Discussion

The results of this study suggest that, in a cohort of women with CIN on cervical and/or cone biopsy, simultaneous multiple HR-HPV infections are common, involving 30% of cases. In multivariate analysis, HPV16 interacted with other HR-HPVs and simultaneous infections were more common than expected for the dyad HPV16 and HPV18 and less than expected for that of HPV16 with HPV33, 51, 52, and 66. In logistic models, simultaneous infections of HPV16 with HPV18 and HPV52 were significantly associated with an excess of high-grade CIN diagnoses and with an overall increased severity of CIN lesions. Among subjects receiving conization, residual disease on ectocervical and endocervical margins was not affected by the presence of simultaneous multiple HR infections. Finally, among women who underwent conization due to CIN confirmation and were followed for a median of 25 months, the recurrence of severe CIN was similar among single and multiple HR infections at entry.

The rate of simultaneous multiple HR-HPVs of the present study is comparable to the 20–56% rate of multiple infections reported in the literature either in cytologic or histological series [1–7]. In multivariate analyses, the rate of coinfection was heavily influenced by the severity of CIN. False positive detection of multiple HPV infections (cross hybridization) is more frequent with low sensitive molecular probes and for coinfections with HPVs with the same *α*-species which share a large percent of the identity of the L1 gene DNA [6–8]. We used a highly sensitive molecular assay in our study, and the diagnosis of CIN on cytology/biopsy was confirmed by conization. In addition, with the only exception of HPV52 and HPV33 which belong to the same species, HPV16 (species *α*-9) interacted with *α*-7 (HPV18), *α*-5 (HPV51), and *α*-6 (HPV66) species. The rates of HPV coinfection of different *α*-species can be influenced by the type of population studied and geographic differences derived from genetic mutation of viral genome consequent to human migration and evolution [3], age, and cytologic and histological results [4, 6, 7, 9]. Previous histological microdissection approaches have initially suggested that any individual histological cervical lesion is provoked by a single virus, thus reducing the pathogenetic importance of HR-HPV interactions [17]. However, recent series suggest that multiple HR-HPVs coexist in about 25% of CIN cases associated with multiple HR-HPVs, underscoring the pathogenetic role of multiple infections [18]. Pairwise association of HPVs in the literature has been studied mainly on cytologic screening samples obtaining varying results [1, 2, 4, 5]. In particular, significant positive interactions have been reported for HPV16 and HPV18 [19, 20] and HPV16 and HPV45 [5] and negative interactions for HPV16 and HPV51 or HPV53 [4], whereas other studies suggest that coinfections among HR-HPV types occur randomly [1, 8]. The different methods used, the sociodemographic variability associated with populations studied, the dissimilarity in the prevalence of various HR-HPVs, and the specimens used (cytology vs histology) are some of the possible reasons for the heterogeneity of these results [8]. The biological mechanisms regulating the dynamics of HPV16 coinfection with other HR-HPV types involve a potential interference in the entry/replication of the viruses, but also in the ability of multiple HR-HPVs to sustain tissue oncogenic transformation in separate lesions or whole tissue sections [17, 18]. Studies in vitro suggest that transfection with other HR-HPVs such as HPV18 or HPV45 of cells already infected with HPV16 produces a suppression of the HPV16 genome replication, potentially reducing the infectivity [13]. Superinfection exclusion is a viral mechanism by which a cell infected by a virus is unable to be infected by a virus of the same or different species [12] This phenomenon has been demonstrated to occur for HPV16 and HPV18 coinfections, and in vitro experiments suggest that HPV16 is able to block HPV18 infection at an early point in time during infection but not in HPV16 persistent cell lines [12]. This suggests that both genomes compete for transcription only in the early phase of infection but are equally efficient in replication in persistent infections [12]. These data support the notion that the rates of coinfection could be significantly influenced by the severity of the lesion, a proxy for persistent infection. Interestingly, our data show that the occurrence of HPV16 coinfection with other HR HPV was uncommon in low-grade CIN increasing with the severity of CIN lesions. According to other studies [4, 21], in our series, interaction between HPV16 and other HPV types was mainly negative confirming the possibility that an existent HPV16 infection could have a suppressive role against a new incident HPV infection.

An essential point in the study of the significance of HR-HPV coinfection is whether multiple infections are clinically relevant, i.e., if coinfection is associated with an increased or reduced risk of CIN or cancer or with an increased risk of progression or recurrence of CIN. Numerous studies have associated overall multiple HR-HPV infections with an increased prevalence and severity of CIN, especially among young women with multiple sexual partners [22] or HIV infected subjects [23]. Multiple HR-HPV infections have also been associated with larger colposcopic lesions [10] and increased risk of progression among subjects with ASCUS and LSIL on cytology [24], and high risk of treatment failure among women with invasive cervical cancer [25]. Overall, results from the present study suggest that there was an excess of CIN 3 among women with multiple infections compared to those with a single infection. Multiple HR-HPV infections at enrollment did not influence the rate of high-grade residual disease on cone examination or the recurrence of high-grade disease during follow-up, suggesting that the role of HPV coinfection on the success of treatment or late recurrence of the disease is limited.

Although by and large HR multiple infections seem to be associated with an increasing severity of HPV-related lesions, the role of HPV type interactions within multiple infections is less studied. A recent study from China [21] suggests that excluding HPV18 and HPV16 single infection was associated with a higher risk of CIN 3+ compared to multiple HPV infection not containing HPV18, implying a negative interaction between HPV16 and negative HPV18 subjects. In the same study, the prevalence of CIN3+ lesions attributable to HPV16 and HPV18 coinfection did not differ from that associated with a single HPV16 infection [21]. The risk of CIN 2 was higher in subjects with coinfection HPV16/HPV18 rather than that associated with HPV18 infection alone. In our study, we found an excess of CIN 3 diagnoses among women with HPV16/18 or HPV16/52 coinfections. The excess number of CIN 3 cases associated with HPV16/18 (*n* = 23) and HPV16/52 (*n* = 28) represent 7.5% of all cases of CIN 3 in this series. These results were confirmed in logistic analyses corrected for potential confounders and by the use of both an ordinal model which captures the relationship between explanatory variables and increasing severity of the outcome, and multinomial models which in turn evaluate the effect of explanatory variables on distinct outcome categories. The main differences between our study and those of Wu et al. [21] are the different genotyping methods and specimens used and the type of population studied. In their study, Wu et al. excluded women less than 30 years of age and used a genotyping method able to distinguish between HPV 16 and HPV 18 infections and it was carried out exclusively on cervical biopsies [21]. In our study, we studied a younger population with a genotyping method that can distinguish between 32 HPV types; in addition, we used conization samples as confirmatory diagnoses. Since the diagnosis of HPV coinfections is heavily influenced by age (being higher in younger women), by the type of genotyping system and by the severity of the cervical disease as diagnosed by biopsy or conization [2, 7, 8], the differences between the two studies seem plausible.

Compared to other investigations evaluating the occurrence and clinical significance of multiple HR-HPVs, the main strengths of this study were the homogeneity of population and the methods used, the histological diagnoses of CIN, and the evaluation of the effect of multiple HR-HPVs on the adequacy of treatment (residual disease on cone) and subsequent recurrence of high-risk disease. On the other hand, the observational nature of the study, a single hospital recruitment center, and the lack of a negative control population are the main limitations of this series.

## 5. Conclusion

In conclusion, the results of this study suggest that, overall, coinfection with multiple HPV types is associated with an excess number of histologically confirmed CIN3 cases. In log-linear analysis, HPV16 interacted positively with HPV18 and negatively with HPV33, 51, 52, and 66 supporting the notion that HPV16 interacts mostly negatively with other HR-HPVs in CIN lesions. On the other hand, there was an excess (though modest in absolute numbers) of CIN3 cases among women coinfected with HPV16 and HPV18 or HPV52 suggesting that some interactions, albeit uncommon, can increase the risk of CIN. Finally, multiple infections did not influence the rates of residual disease on cone examination or the rate of recurrence of high-grade CIN during follow-up confirming the fact that the effect of HR-HPV interaction is limited on the natural history of CIN.

## Figures and Tables

**Table 1:** Sociodemographic and virological data according to the severity of cervical intraepithelial neoplasia (CIN) at the enrollment.

	CIN 1 (*n* = 1811)	CIN 2 (*n* = 405)	CIN 3 (*n* = 678)	Invasive cancer (*n* = 91)
	*N*. 1811 (%)	*N*. 405 (%)	*N*. 678 (%)	*N*. 91 (%)
Age (mean ± SD)	36.8 ± 10.4	35.06 ± 9.3	37.33 ± 9.1^*∗*^	43.76 ± 9.1^*∗*#^
Parity	784 (43.3)	176 (43.46)	298 (43.9)	40 (44)
HIV positive	72 (4)	26 (6.42)	47 (6.9)	4 (4.4)
Nonsmokers	1410 (77.8)	322 (79.5)	528 (77.9)	64 (70.3)
<10 cig/day	197 (10.9)	47 (11.6)	85 (12.5)	16 (17.6)
≥10 cig/day	204 (11.3)	36 (8.9)	65 (9.6)	11 (12.1)
*Contraceptive use*				
No	933 (51.5)	212 (52.3)	382 (56.3)	42 (46.1)
Barrier	155 (8.6)	32 (8)	41 (6)	10 (11)
Hormonal	702 (38.8)	158 (39)	249 (36.7)	37 (40.7)
IUD	21 (1.2)	3 (0.7)	6 (0.9)	2 (2.2)
*Pap smear results*				
ASCUS	584 (33.2)	125 (30.9)	148 (21.8)	5 (5.5)
LSIL	1087 (60.0)	120 (29.6)	212 (31.3)	10 (11)
HSIL-ASCH	127 (7.0)	141 (34.8)	253 (37.3)	28 (30.8)
AGUS	9 (0.5)	12 (3)	25 (3.7)	11 (12.1)
Cancer	4 (0.2)	7 (1.7)	40 (5.9)	37 (40.7)
HPV 16	438 (24.2)	129 (31.8)	278 (41)	43 (47.2)^*∗*#^
HPV 18	113 (6.2)	33 (8.1)	68 (10.0)	10 (11)^*∗*#^
HPV 31	223 (12.3)	66 (16.3)	133 (19.6)	9 (9.9)^*∗*#^
HPV 33	62 (3.4)	26 (6.4)	40 (5.9)	3 (3.3)^#^
HPV 35	42 (2.3)	10 (2.5)	21 (3.1)	1 (1.1)
HPV 39	116 (6.4)	19 (4.7)	48 (7.1)	9 (9.9)
HPV45	40 (2.2)	18 (4.4)	25 (3.7)	1 (1.1)^#^
HPV 51	226 (12.5)	51 (12.6)	85 (12.5)	10 (11)
HPV 52	357 (19.7)	87 (21.5)	187 (27.6)	25 (27.5)^*∗*#^
HPV 56	79 (4.4)	17 (4.)	32 (4.7)	7 (7.7)
HPV 58	55 (3.0)	19 (4.7)	23 (3.4)	2 (2.2)
HPV 66	123 (6.8)	28 (6.9)	32 (4.7)	11 (12.1)^*∗*#^
*Class of risk*				
Negative/undetermined/LR				
HR single	508 (28.1)	57 (14.1)	58 (8.6)	7 (7.7)
HR multiple	849 (46.9)	212 (52.3)	328 (48.4)^*∗*^	51 (56)^*∗*^
	454 (25.1)	136 (33.6)	292 (43.1)^*∗*^	33 (36.3)^*∗*#^

^*∗*^ *p* < .05 compared to CIN 1, ^#^*p* for trend <05. IUD: intrauterine device; ASCUS: atypical squamous cells of undetermined significance; LSIL: low-grade squamous intraepithelial lesion; HSIL: high-grade squamous intraepithelial lesion; ASCH: atypical squamous cells cannot exclude high-grade intraepithelial lesion: AGUS: atypical glandular cells of undetermined significance; HPV: human papillomavirus; LR: low-risk; HR: high-risk.

**Table 2:** Significant interactions between HPV type 16 and other high-risk HPVs in a cohort of 2985 subjects with cervical intraepithelial neoplasia (CIN) or invasive cervical cancer.

Model coefficients							
Predictor	Estimate	SE	*Z*	*p*	Rate ratio	95% confidence interval	
						Lower	Upper
*CIN 1*							
HPV16/HPV 52	-0.461	0.1505	3.06	0.002	0.6308	0.4697	0.8471

*CIN 2 +*							
HPV 16/HPV 18	0.830	0.2019	4.11	<0.001	2.2926	0.4447	3.4057
HPV 16/HPV 31	−0.485	0.1658	2.93	0.003	0.6155	0.2847	0.8519
HPV 16/HPV 51	−0.849	0.2078	4.08	<0.001	0.4279	0.4953	0.6430
HPV 16/HPV 52	−0.423	0.1426	2.97	0.003	0.6551	0.1666	0.8663
HPV 16/HPV 53	−1.243	0.2803	4.43	<0.001	0.2886	0.1252	0.4999
HPV 16/HPV 66	−1.398	0.3467	4.03	<0.001	0.2470	0.1252	0.4873

*Overall*							
HPV 16/HPV 18	0.683	0.1410	4.84	<0.001	1.9795	0.3494	2.6095
HPV 16/HPV 33	−0.609	0.2260	2.69	0.007	0.5441	0.5168	0.8474
HPV 16/HPV 51	−0.404	0.1306	3.10	0.002	0.6676	0.6295	0.8623
HPV 16/HPV 52	−0.255	0.1061	2.40	0.016	0.7751	0.3164	0.9542
HPV 16/HPV 66	−0.768	0.1954	3.93	<0.001	0.4641	1.5434	0.6808

**Table 3:** Rates of cervical intraepithelial neoplasia (CIN) diagnoses according to the interaction between HPV type 16 and HPV33, 51, 52, and 66.

	CIN 1	CIN 2	CIN 3	Invasive cancer
	(*n*) 1811 (%)	(*n*) 405 (%)	(*n*) 678 (%)	(*n*) 91 (%)
Single HPV 16 (*n* = 491)	246 (13.6)^#^	75 (18.5)	140 (20.6)^*∗*^	30 (33)^*∗*^
HPV16 and HPV18 negative (*n* = 1972)	1296 (71.6)^*∗*^	256 (63.2)^#^	377 (55.6)^#^	43 (47.2)^#^
HPV16 and HPV18 positive (*n* = 99)	36 (2)^#^	13 (3.2)	45 (6.6)^*∗*^	5 (5.5)
Single HPV 18 (*n* = 65)	42 (2.3)	9 (2.2)	11 (1.6)	3 (3.3)
Multiple HPV 16 (no HPV 18) *n* = 298	156 (8.6)^#^	41 (10.1)	93 (13.7)^*∗*^	8 (8.9)
Multiple HPV 18 (no HPV 16) *n* = 60	35 (1.9)	11 (2.7)	12 (1.8)	2 (2.2)
HPV16 and HPV33 negative (*n* = 1991)	1325 (73.2)	253 (62.5)	368 (54.3)	45 (49.4)
HPV 16 and HPV 33 positive (*n* = 25)	14 (0.8)	3 (0.7)	8 (1.3)	0 (−)
Single HPV 33 (*n* = 57)	23 (1.3)	16 (3.95)	16 (2.4)	2 (2.2)
Multiple HPV 16 (no HPV 33) (*n* = 375)	178 (9.8)^#^	51 (12.6)	130 (19.2)	16 (17.6)
Multiple HPV 33 (no HPV 16) (*n* = 49)	25 (1.4)	7 (1.7)	16 (2.4)	1 (1.1)
HPV16 and HPV51 negative (*n* = 1565)	1190 (65.7)^#^	236 (58.3)	335 (49.4)^#^	40 (44)^#^
HPV16 and HPV51 positive (*n* = 85)	52 (2.9)	11 (2.7)	20 (2.9)	2 (2.2)
Single HPV 51 (*n* = 156)	97 (5.4)	23 (5.7)	32 (4.7)	4 (4.4)
Multiple HPV 16 (no HPV 51) (*n* = 312)	140 (7.7)^#^	43 (10.6)	118 (17.4)^*∗*^	11 (12.1)
Multiple HPV 51 (no HPV 16) (*n* = 131)	77 (4.2)	17 (4.2)	33 (4.9)	4 (4.4)
HPV16 and HPV52 negative (*n* = 1598)	1080 (59.6)^#^	209 (51.6)	278 (41)^*∗*^	31 (34.1)^*∗*^
HPV16 and HPV52 positive (*n* = 157)	64 (3.5)^#^	20 (5)	65 (9.6)^*∗*^	8 (8.8)
Single HPV 52 (*n* = 219)	141 (7.8)	31 (7.6)	42 (6.2)	5 (5.5)
Multiple HPV 16 (no HPV 52) (*n* = 240)	128 (7.1)^#^	34 (8.4)	73 (10.8)^*∗*^	5 (5.5)
Multiple HPV 52 (no HPV 16) (*n* = 280)	152 (8.4)	36 (8.9)	80 (11.8)	12 (13.2)
HPV16 and HPV66 negative (*n* = 1836)	1273 (70.3)^#^	252 (62.2)	373 (55)^#^	38 (41.8)^#^
HPV16 and HPV66 positive (*n* = 33)	23 (1.3)	4 (1)	5 (0.8)	1 (1.1)
Single HPV66 (*n* = 88)	56 (3.1)	10 (2.5)	18 (2.6)	4 (4.4)
Multiple HPV 16 (no HPV 66) (*n* = 364)	169 (9.3)^#^	50 (12.3)	133 (19.6)^*∗*^	12 (13.2)^*∗*^
Multiple HPV 66 (*n* = 73)	44 (2.4)	14 (3.5)	9 (1.3)	6 (6.6)

CIN: cervical intraepithelial neoplasia. HPV: human papilloma virus. ^*∗*^*p* < .05 higher than expected rate by the chi-squared test and post-hoc analysis with Bonferroni correction ^#^*p* < .05 lower than expected rate by the chi-squared test and post-hoc analysis with Bonferroni correction.

**Table 4:** Odds ratio (OR) and 95% confidence interval (CI) of CIN and invasive cervical cancer associated with coinfection between HPV type 16 and HPV18 or HPV52.

	Ordered logistic	Multinomial logistic CIN1 vs CIN2–CIN3	Multinomial CIN 1 vs Invasive cancer	Binomial CIN 1-2 vs CIN3+
	OR (95% CI)	OR (95% CI)	OR (95% CI)	
HPV16 and HPV18 negative	Reference	Reference	Reference	Reference
HPV16 and HPV18 positive	3.44 (2.3–5.)^*∗#*^	3.2 (2.1–5)^*∗#*^	4.35 (1.6–11.8)^*∗#*^	3.76 (2.5–5.6)
Single HPV 16	1.98 (1.6–2.)	1.8 (1.5–2.)	3.72 (2.3–6.9)	1.96 (1.6–2.4)
Single HPV 18	1.04 (0.6–1.7)	0.96 (0.6–1.7)	2.17 (0.6–7.4)	1 (0.5–1.8)
Multiple HPV16 (no HPV 18)	1.77 (1.4–2.2)	1.71 (1.3–2.)	1.66 (0.8–3.6)	1.9 (1.5–2.5)
Multiple HPV18 (no HPV 16)	1.36 (0.8–2.2)	1.37 (0.8–2.)	2.2 (0.5–9.6)	1.19 (0.6–2.1)
HPV16 and HPV52 negative	Reference	Reference	Reference	Reference
HPV16 and HPV52 positive	3.2 (2.3–4.4)	2.83 (2–4)	4.74 (2.6–7.5)	3.63 (2.6–5.1)
Single HPV16	2.18 (1.8–2.6)	1.94 (1.6–2.4)	4.42 (6.6–7.5)	2.22 (1.5–2.8)
Single HPV52	1.16 (0.9–1.5)	1.14 (0.8–1.5)	1.44 (0.5–3, 8)	1.16 (0.8–1.65)
Multiple HPV16 (no HPV 52)	1.85 (1.4–2.4)	1.82 (1.4–2.4)	1.51 (0.6–4)	2 (1.5–2.7)
Multiple HPV 52 (no HPV 16)	1.87 (1.4–2.4)	1.68 (1.3–2.2)	3.12 (1.5–6.3)	2.1 (1.6–2.7)

^*∗*^ *p* < 0.05pcompared to single HPV16 infection. ^#^*p* < 0.05 compared to multiple HPV16 without HPV18. CIN: cervical intraepithelial neoplasia. HPV: human papillomavirus. Odds ratio (OR) and 95% confidence interval (CI) were obtained including outcomes either as ordered, multinomial (CIN vs CIN2-3, invasive cancer) or binary (CIN3+ vs CIN1–CIN2). Exposure variables included age (continuous), HIV status (yes, no), parity (yes, no), cigarette smoking at entry (no, <10 cig/day, ≥10 cig/day), contraception use (no, hormonal, barrier, intrauterine device), and interacting HPV.

## Data Availability

The data used to support the study are available from the corresponding author upon request.

## Supplementary Materials

Supplementary Materials Table 5: HPV type 16 confection with either HPV18, HPV33, HPV51, HPV52, or HPV66 and residual disease after conization. *Legend*. HPV: human papillomavirus. CIN: cervical intraepithelial disease. Figure 1: flow chart of the cases recruited and follow-up. Legend: FU: follow-up, ASCUS: atypical squamous cells of undetermined significance; LSIL: low-grade squamous intraepithelial lesion; LEEP: loop electroexcision procedure.
