# Supplementary material for: Clinical Significance of the Interaction between Human Papillomavirus (HPV) Type 16 and Other High-Risk Human Papillomaviruses in Women with Cervical Intraepithelial Neoplasia (CIN) and Invasive Cervical Cancer
Source: J Oncol. 2020 Oct 26;2020:6508180. doi: 10.1155/2020/6508180 (PMC7648694; doi:10.1155/2020/6508180)
Supplement: Supplementary Materials — Table 5: HPV type 16 confection with either HPV18, HPV33, HPV51, HPV52, or HPV66 and residual disease after conization. Legend. HPV: human papillomavirus. CIN: cervical intraepithelial disease. Figure 1: flow chart of the cases recruited and follow-up. Legend: FU: follow-up, ASCUS: atypical squamous cells of undetermined significance; LSIL: low-grade squamous intraepithelial lesion; LEEP: loop electroexcision procedure. [file 6508180.f1.zip › 6508180.f1/fgure 1.docx]

Cervical biopsy

Cohort of patients

enrolled in the study

3601

Yes

2

No

2

yesyes

yesyes

3228 patients

373 patients

excluded

excluded

48

LSIL

1090 patients

FU 6 months

Yes

2

No

2

1301 patients

12 Cervical cancer stage>Ib

231

Negative

biopsy

325

ASCUS

1684 patients

yesyes

yesyes

Leep/conization

Patients

considered in the study

2985
