# Supplementary material for: Clinical Significance of the Interaction between Human Papillomavirus (HPV) Type 16 and Other High-Risk Human Papillomaviruses in Women with Cervical Intraepithelial Neoplasia (CIN) and Invasive Cervical Cancer
Source: J Oncol. 2020 Oct 26;2020:6508180. doi: 10.1155/2020/6508180 (PMC7648694; doi:10.1155/2020/6508180)
Supplement: Supplementary file 3 [file 6508180.f1.zip › 6508180.f1/table 5.odt]

|  | Ectocervical Residual Disease | Endocervical Residual Disease |
| --- | --- | --- |
|  | Negative n=888 (%) | CIN1 n= 168 (%) | CIN 2-3 n=245 (%) | Negative n= 1120 (%) | CIN1 n= 83 (%) | CIN 2-3 n= 98 (%) |
| HPV16/HPV 18 negative | 555 (62.5) | 109 (64.9) | 137 (55.9) | 692 (61.8) | 52 (62.7) | 57 (58.2) |
| HPV16/HPV18 positive | 41 (4.6) | 5 (3) | 13 (5.3) | 52 (4.6) | 1 (1.2) | 6 (6.1) |
| Single HPV 16 | 152 (17.1) | 33 (19.6) | 47 (19.2) | 201 (17.9) | 18 (21.7) | 13 (13.3) |
| Single HPV 18 | 22 (2.5) | 2 (1.2) | 3 (1.2) | 23 (2.1) | 3 (3.6) | 1 (1) |
| Multiple HPV 16 | 96 (10.8) | 17 (10.1) | 38 (15.5) | 123 (11) | 8 (9.6) | 20 (20.4) |
| Multiple HPV 18 | 22 (2.5) | 2 (1.2) | 7 (2.9) | 29 (2.6) | 1 (1.2) | 1 (1) |
| HPV16/HPV51 negative | 510 (57.4) | 103 (61.3) | 129 (52.7) | 641 (57.2) | 50 (60.2) | 51 (52) |
| HPV 16/HPV51 positive | 26 (2.9) | 3 (1.8) | 6 (2.4) | 30 (2.7) | 3 (3.6) | 2 (2) |
| Single HPV 16 | 152 (17.1) | 33 (19.6) | 47 (19.2) | 201 (17.9) | 18 (21.7) | 13 (13.3) |
| Single HPV 51 | 46 (5.2) | 5 (3) | 9 (3.7) | 52 (4.6) | 5 (6) | 3 (3.1) |
| Multiple HPV 16 | 111 (12.5) | 19 (11.3) | 45 (18.4) | 145 (12.9) | 6 (7.2) | 24 (24.5) |
| Multiple HPV 51 | 43 (4.8) | 5 (3) | 9 (3.7) | 51 (4.6) | 1 (1.2) | 5 (5.1) |
| HPV16/HPV52   negative | 438 (49.3) | 87 (51.8) | 103 (42) | 550 (49.1) | 43 (51.8) | 35 (35.7) |
| HPV 16/HPV52 positive | 56 (6.3) | 7 (4.2) | 26 (10.6) | 73 (6.5) | 4 (4.8) | 12 (12.2) |
| Single HPV 16 | 152 (17.1) | 33 (19.6) | 47 (19.2) | 201 (17.9) | 18 (21.7) | 13 (13.3) |
| Single HPV 52 | 69 (7.8) | 12 (7.1) | 20 (8.2) | 83 (7.4) | 7 (8.5) | 11 (1.2) |
| Multiple HPV 16 | 81 (9.1) | 15 (8.9) | 25 (10.2) | 102 (9.1) | 5 (6) | 14 (14.3) |
| Multiple HPV 52 | 92 (10.4) | 14 (8.3) | 24 (9.8) | 111 (9.9) | 6 (7.2) | 13 (13.3) |
| HPV16/HPV66 negative | 552 (62.2) | 104 (61.9) | 137 (55.9) | 684 (61.1) | 52 (62.7) | 57 (58.2) |
| HPV 16/HPV66 positive | 6 (0.7) | 3 (1.8) | 2 (0.8) | 8 (0.7) | 2 (2.4) | 1 (1) |
| Single HPV 16 | 152 (17.1) | 33 (19.6) | 47 (19.2) | 201 (17.9) | 18 (21.7) | 13 (13.3) |
| Single HPV 66 | 22 (2.5) | 4 (2.4) | 7 (2.9) | 29 (2.6) | 3 (3.6) | 1 (1) |
| Multiple HPV 16 | 131 (14.8) | 19 (11.3) | 49 (20) | 167 (14.9) | 7 (8.4) | 25 (25.5) |
| Multiple HPV 66 | 25 (2.8) | 5 (3) | 3 (1.2) | 31 (2.8) | 1 (1.2) | 1 (1) |
| HPV16/HPV33 negative | 703 (79.2) | 139 (82.7) | 190 (77.6) | 891 (79.6) | 71 (85.5) | 70 (71.4) |
| HPV 16/HPV33 positive | 16 (1.8) | 3 (1.8) | 4 (1.6) | 20 (1.8) | 0 (-) | 3 (3.1) |
| Single HPV 16 | 31 (3.5) | 10 (6) | 9 (3.7) | 43 (3.8) | 3 (3.6) | 4 (4.1) |
| Single HPV 33 | 14 (1.6) | 4 (2.4) | 4 (1.6) | 17 (1.5) | 4 (4.8) | 1 (1) |
| Multiple HPV 16 | 19 (2.1) | 2 (1.2) | 5 (2) | 24 (2.1) | 0 (-) | 2 (2) |
| Multiple HPV 33 | 105 (11.8) | 10 (6) | 33 (13.5) | 125 (11.2) | 5 (6) | 18 (18.4) |
